# Solution Structure and Rpn1 Interaction of the UBL Domain of Human RNA Polymerase II C-Terminal Domain Phosphatase

**DOI:** 10.1371/journal.pone.0062981

**Published:** 2013-05-07

**Authors:** Ji-Hye Yun, Sunggeon Ko, Chung-Kyung Lee, Hae-Kap Cheong, Chaejoon Cheong, Jong-Bok Yoon, Weontae Lee

**Affiliations:** 1 Department of Biochemistry, College of Life Science and Biotechnology, Yonsei University, Seoul, Korea; 2 Division of Magnetic Resonance, Korea Basic Science Institute (KBSI), Ochang, Cheongwon, Chungbuk, Korea; National Institute for Medical Research, Medical Research Council, United Kingdom

## Abstract

The ubiquitin-like modifier (UBL) domain of ubiquitin-like domain proteins (UDPs) interacts specifically with subunits of the 26 S proteasome. A novel UDP, ubiquitin-like domain-containing C-terminal domain phosphatase (UBLCP1), has been identified as an interacting partner of the 26 S proteasome. We determined the high-resolution solution structure of the UBL domain of human UBLCP1 by nuclear magnetic resonance spectroscopy. The UBL domain of hUBLCP1 has a unique β-strand (β3) and β3-α2 loop, instead of the canonical β4 observed in other UBL domains. The molecular topology and secondary structures are different from those of known UBL domains including that of fly UBLCP1. Data from backbone dynamics shows that the β3-α2 loop is relatively rigid although it might have intrinsic dynamic profile. The positively charged residues of the β3-α2 loop are involved in interacting with the C-terminal leucine-rich repeat-like domain of Rpn1.

## Introduction

An enzymatic cascade composed of the enzymes E1, E2, and E3 conjugates ubiquitin to target proteins, followed by translocation to the 26 S proteasome, where ubiquitinated protein is removed by proteolysis [1]. Despite low sequence homology, ubiquitin-like proteins are classified as ubiquitin-like modifiers (UBLs) and ubiquitin-like domain proteins (UDPs) [2]. UBLs, such as NEDD8, SUMO, and FAT10, modify target proteins in a manner similar to ubiquitinylation [3]. UDPs, including Rad23, the human homolog of Rad23 (HHR23), Dsk2, ubiquilins 1–4 (human homologs of Dsk2), and parkin, bind to the 26 S proteasome in a UBL domain-dependent manner [4], [5]. The UBL domain of UDPs interacts specifically with subunits of the 26 S proteasome. Rad23 and Dsk2 preferentially interact with the Rpn1/S2 subunit. UDPs have been implicated in neurodegenerative diseases caused by dysfunction of the ubiquitin proteasome system [6]. Recently, a novel UDP protein, ubiquitin-like domain-containing C-terminal domain phosphatase (UBLCP1) has been identified [7]. UBLCP1 consists of two independent domains, UBL and a phosphatase domain. It has been proposed that UBLCP1 directly interacts with Rpn1 of the 26 S proteasome through the UBL domain and that it serves as a transcription regulator via the phosphatase domain [8], [9], [10]. UBLCP1 also inhibits proteasome activity, dephosporylating the 26 S proteasome [10]. The crystal structure of *Drosophila* UBLCP1 shows that the UBL domain and the phosphatase domain are connected by a flexible linker and that the UBL domain has a β-grasp fold with four β-strands and two α-helices [10]. Because UBLCP1 has diverse functions related to regulation of the phosphorylation state of the 26 S proteasome, it is of the essence to understand the detailed interactions between UBLCP1 and Rpn1 of the proteasome component. We determined the solution structure of the UBL domain of human UBLCP1 (hUBLCP1). Structural information indicated detailed intermolecular interactions between the UBL domain and Rpn1 on the atomic scale. Nuclear magnetic resonance (NMR) data revealed that the secondary structure of the UBL domain of hUBLCP1 differs from that of known UBL domains, especially β3 and β4. In addition, the structure of the UBL domain of hUBLCP1 is dramatically different from that of the fly UBLCP1 [10]. Interestingly, the positively charged clamp formed by the unique β3-α2 loop of the UBL domain of hUBLCP1 serves as a key motif in the interaction with Rpn1.

## Materials and Methods

### Cloning, Expression, and Purification of Human UBLCP1 and Rpn1

Polymerase chain reaction (PCR) products of both hUBLCP1 and Rpn1 were amplified from a *Homo sapiens* cDNA library. All sense primers encoded the recognition site of tobacco etch virus protease (ENLYFQG) for the clearance of affinity tags. For both the UBLCP1 and UBL domain (UBLCP1^1–81^), sense primers and antisense primers were designed as previously described [8], and amplified PCR products were ligated into the pGEX 4T-1 vector (Amersham Pharmacia Biotech, Uppsala, Sweden). The sense and antisense primers for three Rpn1 constructs, Rpn1^1–908^, Rpn1^394–568^, and Rpn1^640–772^ incorporated *Bam*HI and *Xho*I restriction enzyme sites, and PCR products were ligated into the pET32a vector. The plasmids were transformed into *E. coli* BL21 (DE3) cells for overexpression. The transformed cells were induced by 0.1 mM isopropyl β-D-thiogalactopyranoside at an OD_600_ of 0.6. All purified proteins were applied to a size exclusion chromatography column (Amersham Pharmacia Biotech) to improve protein purity and exchange the buffer solution.

### Immunoprecipitation and in vitro GST Pull-down Assay

Cells were derived from HeLa Tet-Off (Clontech, Pala Alto, CA, USA) cells. EBNA-1 cells were transfected with pYR-HA-UBLCP1 and pYR-FLAG-RPN1. After 36 hours, cells were harvested and lysed in buffer solution consisting of 50 mM Tris-HCl (pH 7.5), 150 mM NaCl, 1 mM EDTA, 1 mM dithiothreitol, 0.2 mM phenylmethanesulfonyl fluoride, and 1.0% NP-40. The cell lysate was mixed with anti-FLAG or anti-hemagglutinin (HA) antibody-conjugated resin in 0.1% NP-40 for 4 hours at 4°C. Antibody resin was further washed with binding buffer containing 0.1% NP-40. The immune complexes were eluted in 20 mM Tris-HCl (pH 8.0) containing 2% sodium dodecyl sulfate. Immunoprecipitated proteins were detected using FLAG (Sigma, St Louis, MO, USA) and HA (Babco, Richmond, CA, USA) antibodies. The UBL domains (UBLCP1^1–81^) of hUBLCP1, Rpn1 regulatory subunit 1 (Rpn1^394–568^), and Rpn1 regulatory subunit 2 (Rpn1^640–772^) were used for the pull-down assay. GST-fused UBLCP1^1–81^ was loaded onto glutathione-Sepharose 4B resin and washed with lysis buffer. The TRX-His^6^-fused Rpn1^394–568^ and Rpn1^640–772^ were loaded onto the glutathione-Sepharose 4B resin preloaded with UBLCP1^1–81^. After incubation for 1 hour at 25°C, the resin was washed and proteins were eluted using elution buffer containing 10 mM reduced glutathione. Samples were analyzed at each step by 15% sodium dodecyl sulfate polyacrylamide gel electrophoresis.

### NMR Spectroscopy and Structure Calculations

To obtain the ^13^C/^15^N- and ^15^N-labeled UBL domains of hUBLCP1, cells were cultured in M9 media containing 99% ^15^NH_4_Cl or 99% ^15^NH_4_Cl and 99% ^13^C-D-glucose (Cambridge Isotope Inc., Andover, MA, USA). Purified UBL domain was concentrated to 1.5 mM with an Amicon Ultra-15 concentration device (Millipore, Bedford, MA, USA). All NMR experiments were performed on Bruker DRX 500 MHz and Bruker Avance 900 MHz spectrometers equipped with a cryoprobe at 25°C. The ^15^N-edited two-dimensional (2D) HSQC, HNCACB, CBCA(CO)NH, HNCA, HNCO, HBHA(CO)NH, and HCCH-TOCSY experiments were performed for resonance assignments of backbone and side-chain atoms [11]. To obtain nuclear Overhauser effect (NOE) constraints for structure calculations, ^15^N-edited and ^13^C-edited three-dimensional (3D) NOESY experiments with two mixing times (τ = 100 and 150 ms) were performed. All NMR data were processed using NMRPipe [12] and analyzed using the SPARKY program. The in-phase-anti-phase (IPAP) experiment for residual dipolar coupling (RDC) measurements was performed using polyacrylamide gels prepared as described by Sass *et al*. [13]. The 6% polyacrylamide gels were made in a 5-mm inner-diameter tube, and dried after 3 days of dialysis. The ^15^N-labeled UBLCP1^1–81^ (300 µl) was incorporated in the dried gel and placed in a Shigemi NMR tube, and the gel was compressed by the plunger. The ^1^D_NH_ dipolar coupling constants in both isotropic and anisotropic media were measured using the 2D IPAP experiments [14] on the Bruker DRX 500 spectrometer. The RDC constants were calculated from the difference of the ^1^J_NH_ splitting in isotropic and anisotropic media. The alignment tensor was calculated using REDCAT software [15].

NMR titration experiments for analysis of interaction of ^15^N-labeled UBLCP1^1–81^ with Rpn1^394–568^ were conducted using a Bruker DRX 500 MHz equipped with a cryoprobe. Different molar ratios of UBLCP1^1–81^ and Rpn1^394–568^ (1∶0.5, 1∶1, 1∶2) were used for the NMR titration [16]. The average values of the chemical shift changes were analyzed using the equation, △δ_AV_ = ((△δ_1H_)^2^+ (0.2×△δ_15N_)^2^)^1/2^. The symbols △δ_AV_, △δ_1H_, and △δ_15N_ designate average chemical shifts,^ 1^H chemical shifts, and ^15^N chemical shift changes, respectively [17].

Structure calculations were performed using the CYANA 2.1 program [18] installed on a 16-node Linux cluster computer. The alignment tensor was used during the structure refinement procedure. From the RDC, Da and R values were calculated as 1.448±0.015 and 0.622±0.016, respectively. The value of the Q-factor after back calculations was determined as 0.34. A total of 2080 NOE constraints, consisting of 1009 short-range NOEs (|i–j| ≤1), 365 medium-range NOEs (1< |i–j| <5), 706 long-range NOEs (|i–j| ≥5), and 100 angle constraints were used for structure calculations [19]. The PROCHECK program was used for structure evaluation [20] and the MOLMOL, NACCESS, and Pymol programs were used for structural analyses [21].

### Backbone Dynamics

Dynamics experiments were performed as previously described [22]. Longitudinal (T_1_) and transverse (T_2_) relaxation data for backbone amide protons were collected using different relaxation times, T_1_ = 0.05, 0.15, 0.3, 0.5, 0.7, 1, and 1.5 sec and T_2_ = 0.02, 0.04, 0.06, 0.08, 0.1, 0.12, and 0.14 sec. R_1_ and R_2_ decay rates were calculated using the exponential decay function in the CurveFit program (http://cpmcnet.columbia.edu/dept/gsas/biochem/labs/palmer/software/curvefit.html). The steady-state heteronuclear NOE (XNOE) experiments [22], [23] were performed using a relaxation delay of 3 sec, and XNOE values were calculated from peak heights of unsaturated and saturated NOE spectra using the equation σNOE/NOE = [(σIsat/Isat)^2^+(σIunsat/Iunsat)^2^]^1/2^. From the relaxation parameters, τ_m_ and rotational diffusion tensor were calculated. Order parameter (S^2^) and conformational exchange terms (R_ex_) were determined using the FASTModelfree program [24].

### Isothermal Titration Calorimetry

VP-ITC system (MicroCal) was used for ITC experiments of UBL domain and Rpn1 regulatory subunit 1 at 25°C in NMR buffer. In each titration, 20 µM Rpn1 regulatory subunit 1 in the cell was titrated with 25 injections of 400 µM UBL domain. Each injection was 6 µL. The resulting data were fitted to a one-site binding isotherm using Microcal Origin program for ITC data analysis.

## Results and Discussion

### NMR Structures of the UBL Domain of hUBLCP1

**Figure 1:**
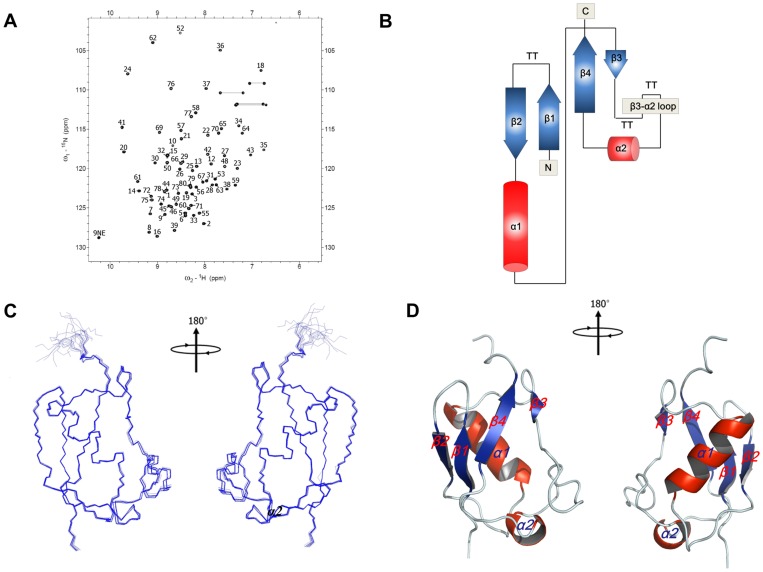
Resonance assignment and solution structure of the UBL domain of hUBLCP1. (A) ^1^H-^15^N HSQC spectrum for the UBL domain was obtained using a Bruker DRX 900 MHz equipped with a cryoprobe. All backbone resonances in HSQC spectrum are labeled. (B) Molecular topology shows that the UBL domain of hUBLCP1 is composed of four β-strands and two α-helices. The unique β3-α2 loop exists in the same location in which β4 is usually found in the other UBLs. (C) The 20 lowest-energy structures were superimposed and fitted for the restraint energy minimization (REM) average structure using backbone atoms, C_α_, N, and CO and the MOLMOL program. (D) Ribbon diagram shows molecular topology of the UBL domain generated by the Pymol program. The four β-strands and two α-helices are labeled on the structure.

All backbone resonance assignments were completed with data from HNCA, CBCACONH, and HNCACB experiments (Fig. 1A). Most of the side chain assignments were made based on 3D HCCH-TOCSY and ^15^N-edited TOCSY-HSQC experiments. Secondary structures were determined from the chemical shift indices (CSIs), NOEs, and ^3^J_HNα_ coupling constant values.

The UBL domain of hUBLCP1 consisted of four short β-strands (β1, β2, β3, and β4) and two α-helices (α1 and α2) (Fig. 1B and Fig. S1). A total of 50 distance geometry structures were used as starting conformers for dynamical simulated annealing calculations. The structures were further refined using the calculated alignment tensor from the IPAP experiment. The 20 lowest-energy structures were selected for the final analysis (Table 1). The final structures were well converged, with a root-mean-square deviation (RMSD) of 0.44±0.13 Å and 0.74±0.11 Å for all backbone and heavy atoms, respectively (Fig. 1C). Especially, a unique β3-α2 loop was well defined by a number of intra-loop NOEs (Fig. 1D and Fig. S2) observed in that region. The average structure was calculated from the geometrical average of the 20 final structures and subjected to restraint energy minimization to correct covalent bonds and angle distortions (Fig. 1D).

**Table 1:** Structural statistics for the UBL domain of hUBLCP1.

	UBL domain of hUBLCP1
**NOE distance restraints (no.)**	
All	2080
Short range (|i–j| ≤1)	1009
Intraresidual NOEs (|i–j| <1)	475
Sequential NOEs (|i–j| = 1)	534
Medium range (1<|i–j| <5 )	365
Long range (|i–j| ≤5)	706
**Dihedral angle restraints (no.)**	
All	100
Φ	49
Ψ	51
**Hydrogen bonds**	22
**RDC restraints**	71
**Mean CYANA target function (Å^2^)**	10.4±0.12
**Mean RMS deviations from the average coordinate (Å)**	
Backbone atoms (N, C_α_, C’, O)	0.44±0.13
Heavy atoms	0.74±0.11
**Ramachandran plot (%)** a	
Most favored regions	82.9
Additional allowed regions	15.1
Generously allowed regions	2.0
Disallowed regions	0

a Ramachandran plot was calculated using the PROCHECK program.

### Structural Comparison with other UBL Domains

**Figure 2:**
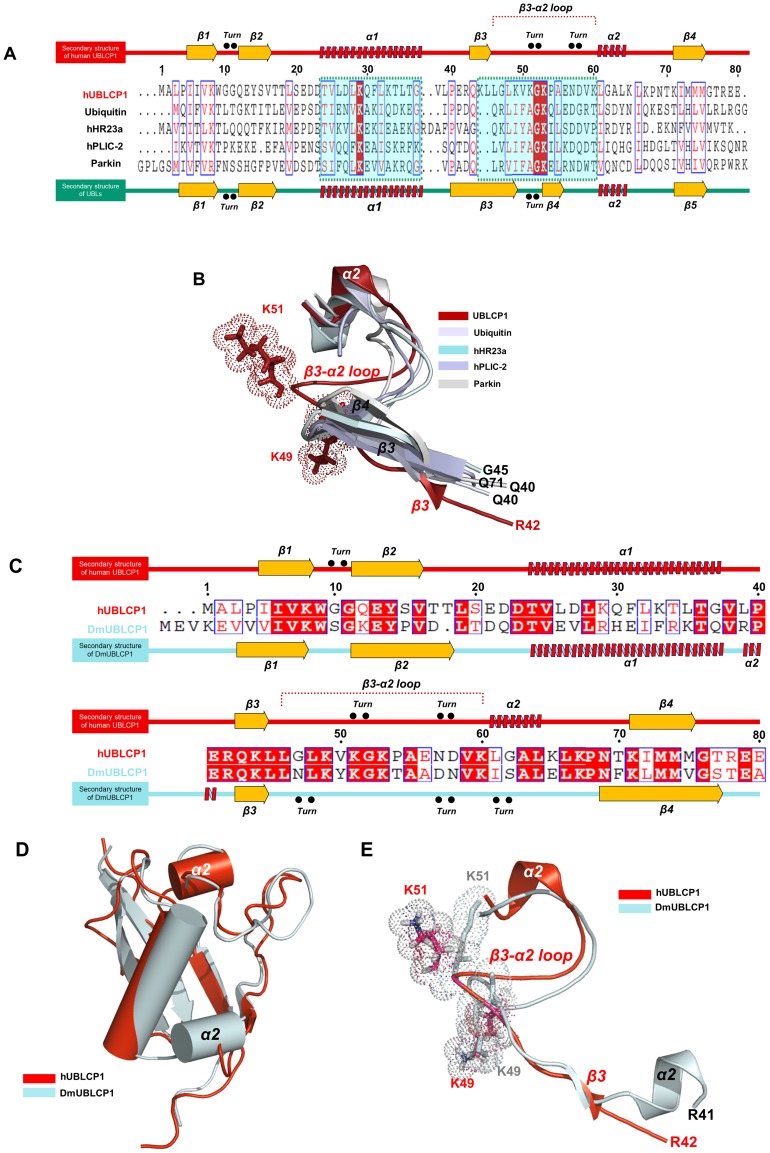
Structural comparison of the UBL domains. (A) Sequence alignment and secondary structures of the UBL domain of hUBLCP1, ubiquitin, hHR23a, hPLIC-2, and parkin. (B) Structural overlay of the β3-α2 loop of the UBL domain (red) of hUBLCP1 with that of ubiquitin (blue-white), hHR23a (pale cyan), hPLIC-2 (light blue), and Parkin (white-gray), respectively. (C) Sequence alignment and secondary structures of the UBL domains of human (hUBLCP1) and *D. melanogaster* (DmUBLCP1) UBLCP1. (D) Structural comparison of the UBL domains of human and *D. melanogaster* UBLCP1. (E) Superposition of the β3-α2 loop regions of the UBL domains of hUBLCP1 (red) and dmUBLCP1 (pale cyan).

The molecular topology of the UBL domain of hUBLCP1 was quite different from that of the canonical UBL domain (Fig. 1B), especially in the organization of the secondary structures (β3 and β4; Fig. 2A and Fig. 2B). The pairwise RMSDs of C_α_ of ubiquitin and the UBL domains of hHR23a, hPLIC-2, and parkin with respect to that of hUBLCP1 were 2.405 Å, 1.391 Å, 3.457 Å, and 3.785 Å, respectively. The fourth strand (β4) in the other UBLs was not observed in hUBLCP1; however, a unique β3-α2 loop was observed in that region (Fig. 1B and Fig. 2A). In addition, unlike other UBL domains, the UBL domain of hUBLCP1 has a short β3, comprising residues Q43, K44, and L45. Most of the unique β3-α2 loop in UBLCP1^1–81^ is exposed to the solvent (Fig. 2B). Very recently, the crystal structure of dmUBLCP1 derived from *Drosophila melanogaster* was reported [10]. Although the UBL domain of dmUBLCP1 has a high percentage of sequence identity (54%) with that of human UBLCP1, dramatic differences between the two structures were observed (Fig. 2C, 2D, and 2E). Surprisingly, two α-helices (α1 and α2) are connected by a short linker in the UBL domain of the dmUBLCP1. This is very unusual because α2 is located far from α1 (next to β3/β4) in most of the UBL domains, including that of hUBLCP1 (Fig. 2A, 2B, and Fig. S1). However, the structural folds in the UBL domains of hUBLCP1 and dmUBLCP1 are very similar, consistent with the backbone RMSD between the two structures of 1.778 Å (Fig. 2D).

### Molecular Interaction between hUBLCP1 and Rpn1

**Figure 3:**
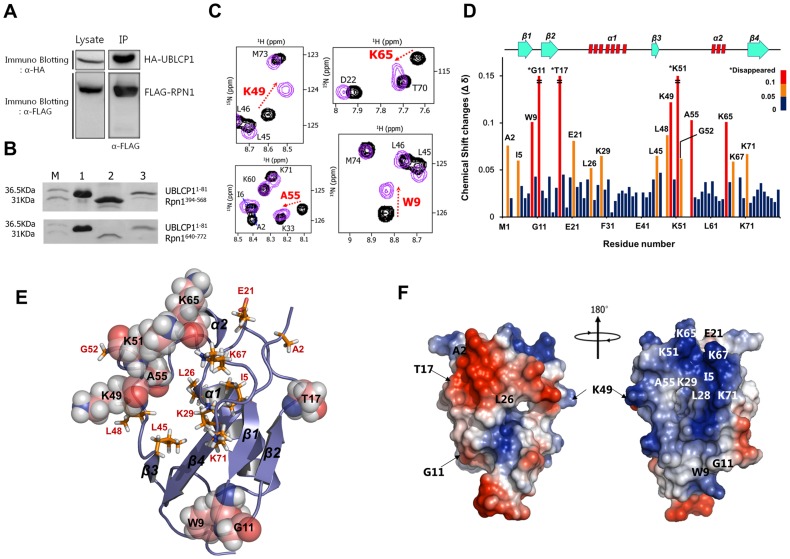
Intermolecular interaction between hUBLCP1 and Rpn1. (A) Immunoprecipitation (IP) of both hUBLCP1 and Rpn1 was performed. Immunoblotting was conducted using HA- and FLAG-antibodies to distinguish the two proteins. IP was performed for the a-FLAG-RPN1 and co-precipitation was confirmed by IB. (B) *In vitro* GST pull-down assay using the GST-fused UBL domain of hUBLCP1 (UBLCP1^1–81^), the TRX-His^6^-fused regulatory subunit 1 (Rpn1^394–568^), and subunit 2 (Rpn1^640–772^). Lane 1 (upper and lower), GST-fused UBL domain of UBLCP1; Lane 2 (upper), TRX-His6-fused Rpn1^394–568^ and (lower), TRX-His6-fused Rpn1^640–772^; Lane 3 (upper and lower), pull-down elution using GST elution buffer containing 10 mM reduced glutathione. (C)^ 15^N-labeled UBL domain was titrated with Rpn1^394–568^. Superimposed 2D ^1^H-^15^N HSQC spectra of UBL domain without Rpn1^394–568^ (black) and with Rpn1^394–568^ (purple). To assess chemical shift perturbations of the UBL domain on Rpn1 binding, titration of 0–2 molar equivalents of Rpn1^394–568^ was performed. Residues K49 and A55 in the β3-α2 loop, K65 in the α2-β4 loop and W9 in the β1-β2 loop are displayed. (D) Chemical shift changes (△δ) of the UBL domain upon Rpn1^394–568^ binding are summarized. Chemical shift perturbations are shown by different colors: blue, 0 ppm<△δ ≤0.05 ppm; orange, 0.05 ppm<△δ ≤0.1 ppm; red, 0.1 ppm<△δ ≤ disappeared. Residues that "disappeared," due to peak broadening, are marked by asterisks and the secondary structures are also displayed. (E) Residues with significant chemical shift changes and less perturbed changes upon Rpn1 binding drawn by the Pymol program are shown by spheres and sticks, respectively. (F) Surface charge model of the UBL domain of the hUBLCP1. Electrostatic surfaces are shown for negative (red), positive (blue), and neutral (white) potential.

Because the structure of the UBL domain of hUBLCP1 is unique, detailed analysis of Rpn1 binding is of the essence in understanding the mechanism underlying the interaction between the two molecules. Using an immunoprecipitation assay, we showed that hUBLCP1 directly interacts with the Rpn1 of the regulatory particle of the 26 S proteasome (Fig. 3A). A recent study suggests that leucine-rich repeat-like domains of Rpn1 recognize the ubiquitin motif [25]. Homology modeling and secondary structure predictions show that Rpn1 has two structural subunits, regulatory subunits 1 (Rpn1^394–568^) and 2 (Rpn1^640–772^) with leucine-rich repeat-like-rich sequences (data not shown). Data from the GST pull-down assay showed that regulatory subunit 1 (Rpn1^394–568^) directly interacts with UBLCP1^1–81^, whereas regulatory subunit 2 (Rpn1^640–772^) of Rpn1 does not (Fig. 3B).

We performed NMR titration experiments to identify binding residues of the UBL domain of hUBLCP1 upon Rpn1 binding in solution. A number of chemical shift perturbations were observed upon Rpn1^394–568^ titration (Fig. 3C and 3D). Most of the resonance perturbations in the UBL domain of hUBLCP1 were found in the residues of the loop regions (i.e., W9, G11, T17, K49, K51, A55, and K65; Fig. 3E and 3F). Interestingly, K49, K51, and A55 in the β3-α2 loop are mainly involved in Rpn1 binding, which suggests the β3-α2 loop is responsible for Rpn1 binding. To determine the binding affinity of the UBL domain of hUBLCP1 for Rpn1^394–568^, we performed isothermal titration calorimetery experiments. The dissociation constant (K_d_) and the enthalpy *(*Δ*H*) were calculated as 1.03×10^−5^±4.13×10^−5^ M and −5.38×10^−4^±8870 cal/mol, respectively.

### Backbone Dynamics

**Figure 4:**
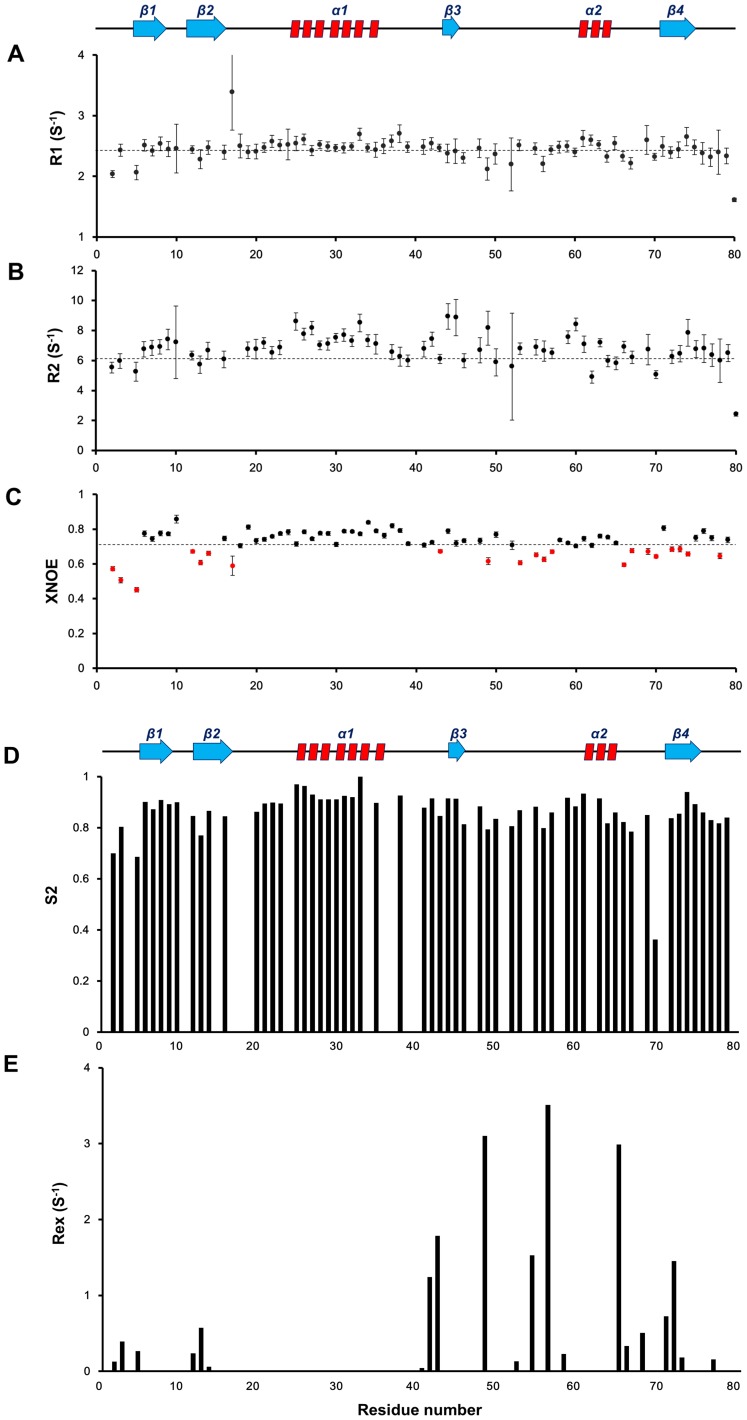
Backbone dynamics of the UBL domain of hUBLCP1. The values for (A) spin-lattice relaxation time (T_1_) and (B) spin-spin relaxation time (T_2_) are shown. (C) Heteronuclear NOE (XNOE) data are displayed. Error values were calculated using the following equation: σNOE/NOE = [(σIsat/Isat)^2^+ (σIunsat/Iunsat)^2^]^1/2^. (D) Calculated order parameter (S^2^) and (E) conformational exchange terms (R_ex_) are shown. All relaxation parameters were determined by the FASTModelfree program.

Although the UBL domain of hUBLCP1 contains the canonical fold of UBL domains, the secondary structures and β3-α2 loop are very distinct. To correlate structure and dynamic properties of the UBL domain of hUBLCP1, we performed NMR backbone relaxation (Fig. 4A and 4B) and ^15^N-^1^H heteronuclear NOE experiments (Fig. 4C). The overall average R1, R2, and NOE values were determined as 2.44±0.13 s^−1^, 6.73±0.61 s^−1^, and 0.71±0.013, respectively. Interestingly, the average R1, R2, and XNOE values of the residues in the β3-α2 loops were determined as 2.36±0.139 s^−1^, 6.825±0.88 s^−1^, and 0.62, indicating that the β3-α2 loop is relatively rigid although it might have intrinsic dynamic profile. The order parameters and conformational exchange supported all dynamic data, with the average value of order parameters calculated as 0.861±0.037 (Fig. 4D). The conformational exchange parameters suggest an evidence of rapid exchange characteristics for some residues in the β3-α2 loop (Fig. 4E).

### Functional Implications

Although UBLCP1 has been classified as a member of the UDP family, it is important to note that the UBL domain of hUBLCP1 has a unique structural feature, which could be related to its specific function. It is well known that UBLs recognize the ubiquitin-associated domain or ubiquitin-interacting motif through their conserved hydrophobic residues [26]. The hydrophobic residues in the β3-β4 loop and β5 of hPLIC-2, hHR23a, and parkin are mainly involved in molecular interactions with their partner proteins [27]–[29]. Our findings suggest that the UBL domain of hUBLCP1 interacts with regulatory subunit 1 of Rpn1 via hydrophilic residues of the unique β3-α2 loop. Therefore, we hypothesize that the unique structure of the UBL domain of hUBLCP1 could be responsible for specific recognition of partner molecules, such as Rpn1. Data from proteomics studies have suggested that the peptidase activity of individual subunits of the murine cardiac 20 S proteasome is enhanced by phosphorylation and that the activity is tightly regulated by protein phosphatase 2A and protein kinase A [30], [31]. The UBL domain of hUBLCP1 interacts with Rpn1 of the regulatory unit of the 26 S proteasome, and hUBLCP1 has been identified as one of the phosphatases functionally integrated with dephosphorylation of regulatory particles in the 26 S proteasome. Therefore, our findings will be directly applicable to future investigation of molecular interactions with the proteasome complex during regulation of the 26 S proteasome through posttranslational modification of regulatory particles.

## Supporting Information

Figure S1 **A summary of NOE connectivity of the UBL domain of hUBLCP1.** (A) Strips of NH-NH NOEs from 3D ^15^N-edited 3D NOESY-HSQC spectrum. Sequential and medium-range NOEs from L61 to L64 are indicative of α-helical structure. Sequential (*d_NN_*) and medium-range NOEs (*d_NN (i, i_*_+2)_) are marked by red and blue lines, respectively. (B) **A** Summary of NOE connectivity and secondary structures predicted by the CSI program. NOEs and consensus CSI values show that the UBL domain of hUBLCP1 consists of two α-helices and four β-strands.
(TIF)

Figure S2 **Short and medium range NOEs for a unique β3-α2 loop region of the UBL domain of hUBLCP1.** (A) Examples of NOE peaks from the 3D ^15^N-edited 3D NOESY-HSQC spectrum. Intra-residue and inter-residue NOEs are shown in blue and red, respectively. (B) A list of observed NOEs in the β3-α2 loop region. NOE intensities are classified as strong (s), medium (m) and weak (w).
(TIF)
