# Supplementary figures and images for: Solution Structure and Rpn1 Interaction of the UBL Domain of Human RNA Polymerase II C-Terminal Domain Phosphatase
Source: PLoS One. 2013 May 7;8(5):e62981. doi: 10.1371/journal.pone.0062981 (PMC3646893; doi:10.1371/journal.pone.0062981)

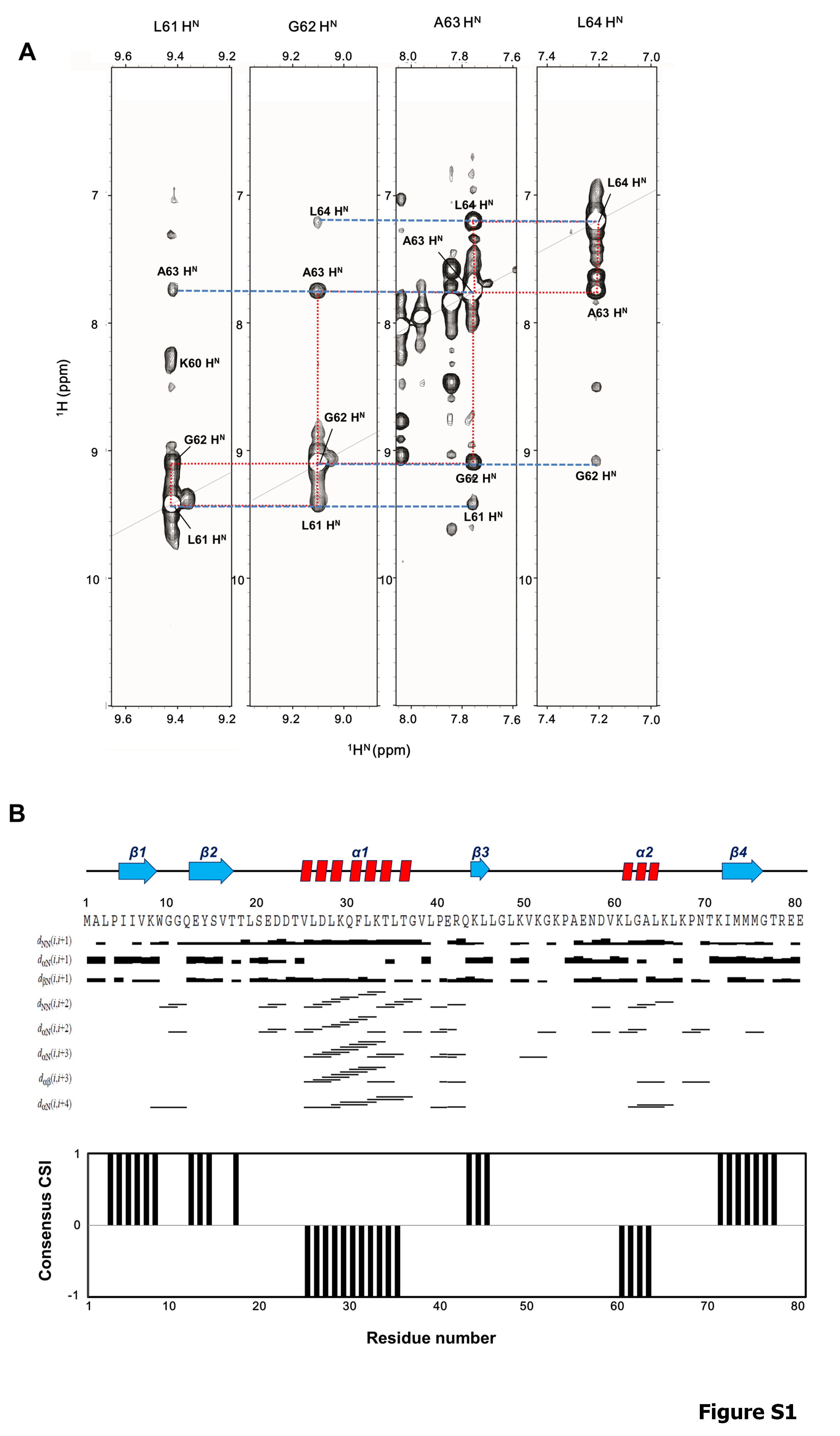

Supplement: Figure S1 — A summary of NOE connectivity of the UBL domain of hUBLCP1. (A) Strips of NH-NH NOEs from 3D 15N-edited 3D NOESY-HSQC spectrum. Sequential and medium-range NOEs from L61 to L64 are indicative of α-helical structure. Sequential (dNN) and medium-range NOEs (dNN (i, i +2)) are marked by red and blue lines, respectively. (B) A Summary of NOE connectivity and secondary structures predicted by the CSI program. NOEs and consensus CSI values show that the UBL domain of hUBLCP1 consists of two α-helices and four β-strands. (TIF) [file pone.0062981.s001.tif]

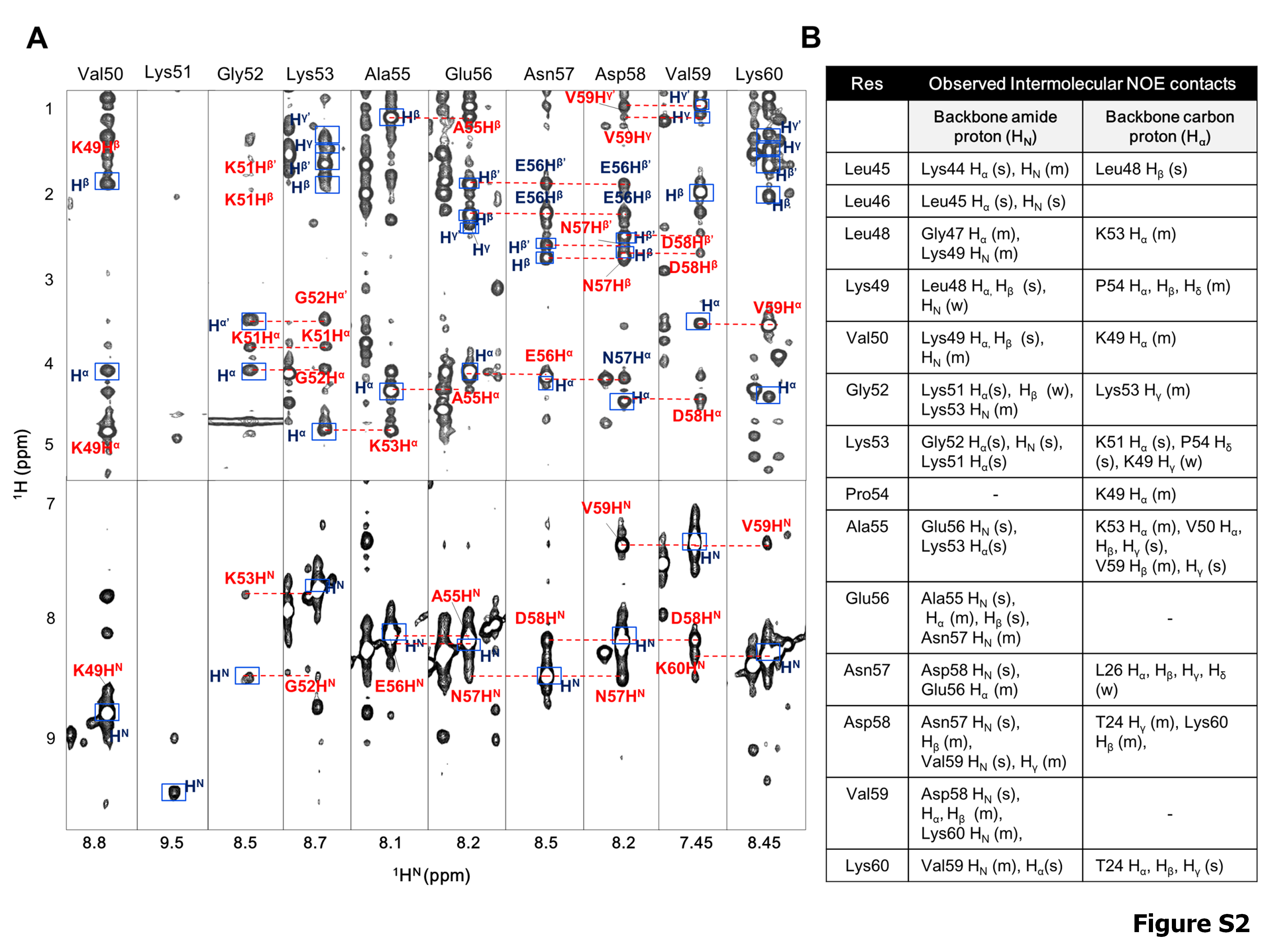

Supplement: Figure S2 — Short and medium range NOEs for a unique β3-α2 loop region of the UBL domain of hUBLCP1. (A) Examples of NOE peaks from the 3D 15N-edited 3D NOESY-HSQC spectrum. Intra-residue and inter-residue NOEs are shown in blue and red, respectively. (B) A list of observed NOEs in the β3-α2 loop region. NOE intensities are classified as strong (s), medium (m) and weak (w). (TIF) [file pone.0062981.s002.tif]
